# Insights Into the Impacts of BRCA Mutations on Clinicopathology and Management of Early-Onset Triple-Negative Breast Cancer

**DOI:** 10.3389/fonc.2020.574813

**Published:** 2021-01-11

**Authors:** Fugui Ye, Min He, Liang Huang, Guantian Lang, Xin Hu, Zhimin Shao, Genhong Di, Ayong Cao

**Affiliations:** ^1^ Key Laboratory of Breast Cancer in Shanghai, Department of Breast Surgery, Fudan University Shanghai Cancer Center, Shanghai, China; ^2^ Department of Oncology, Shanghai Medical College, Fudan University, Shanghai, China; ^3^ Institutes of Biomedical Sciences, Fudan University, Shanghai, China

**Keywords:** early-onset, triple-negative, breast cancer, breast cancer type 1 susceptibility protein, mutation

## Abstract

**Background:**

Little is known regarding the clinicopathologic characteristics, oncologic outcomes, and treatment strategies that could be ascribed to BRCA mutation in early-onset triple-negative breast cancer (eTNBC).

**Methods:**

eTNBC patients who underwent BRCA genetic testing were derived from our clinical database between 2012 and 2018. Differences in clinical features and pathologic characteristics were examined in groups divided by BRCA mutation status, and the contribution of germline mutations in conjunction with treatment modalities to survival outcomes was determined.

**Results:**

Of the 355 qualifying eTNBC patients, 67 (18.87%) were BRCA mutated and 288 (81.13%) were BRCA wild. Overall, median age at diagnosis was 34 years (range, 24–40 years) in the BRCA mutated subgroup and 35 years (range, 21–40 years) in BRCA wild. The majority of clinicopathologic parameters were parallel; however, tumor size (*P* = 0.07) and nuclear grade (*P* =0.08) tend to be more aggressive in the BRCA mutated subgroup. Compared with BRCA wild patients, BRCA mutated patients had a higher likelihood of receiving anthracyclines and taxane-based combination chemotherapy (*P* = 0.04) and tend to be lower tumor burden (*P* =0.01). After approximately 5-year median follow-up, the overall survival (OS) (*P* = 0.021) and breast cancer-specific survival (BCSS) (*P* = 0.004) in BRCA mutated patients were superior to those in their BRCA wild counterparts. Intriguingly, the clinical outcomes were comparable in patients with breast conserving surgery (BCS) regardless of BRCA mutations and in patients with BRCA mutations in spite of surgical schedules.

**Conclusions:**

These results suggest that eTNBC patients with BRCA mutations are prone to better OS and BCSS, which might be largely attributed to more benefit from anthracyclines and taxane-based chemotherapy. The BCS procedure could be a safe alternative surgical option for eTNBC patients with BRCA mutations. Future studies with substantial numbers of participants are urgently needed to validate whether BRCA mutation eTNBC patients are more sensitive to chemotherapy.

## Introduction

Breast cancer incidence increases with age (1). Young age breast cancer, diagnosed before the age of 40 years, is a unique biological and clinical entity and currently represents a top biomedical research priority. Epidemiologically, it is now established knowledge that the proportion of women diagnosed with breast cancer before the age of 35 in the West and before 40 in the East is about 4 and 13%, respectively (1, 2). Accumulating evidence suggests that young age breast cancer is the leading cause of cancer-related deaths of women under the age of 45 years and has been listed as the paramount health burden in developing countries compared with their developed counterparts (3).

Generally, early age of breast cancer onset is considered an indicator of cancer susceptibility genes (4). A substantial proportion of hereditary breast cancer can be attributed to mutations in one of two genes, BRCA1 or BRCA2 (5). The literature has documented that women who inherited a deleterious BRCA mutation suffer a high lifetime risk of developing breast cancer (6–8), with a large-sized prospective study estimating a cumulative incidence of 66 and 61% for BRCA1 and BRCA2 up to the age of 70 years, respectively (6). BRCA mutations are the most common genetic variabilities in breast cancer and closely associated with aggressive clinical and biologic course of breast cancer, especially the triple-negative breast cancer (TNBC) subtype (9, 10). It is estimated that 10% of patients with TNBC present with deleterious germline mutations in BRCA1 or BRCA2 (11). Around 60 to 80% patients carrying a BRCA1 germline mutation are characterized by TNBC phenotype (12), and 15 to 25% TNBC patients of Ashkenazi ethnicity have a BRCA1 mutation (13, 14). Intriguingly, in contrast to BRCA2, BRCA1 mutations are thought to contribute to more cases of early onset breast cancer (15).

BRCA mutation cancers possess a deficiency in homologous recombination repair of DNA double-strand breaks (DSBs), thus causing genomic instability (16, 17). Drugs that induce DSB have shown sensitivity to and promise for BRCA-associated TNBC in a series of clinical trials (18). Many other pathway-specific inhibitors have been investigated to overcome the drawbacks of current treatment options for TNBC in recent years (19). The current screening, recommendations, therapeutic strategies, and even surveillance of BRCA-associated TNBC are in reference to sporadic TNBC. Despite this intensive investigation of the penetrance of BRCA mutations in early onset TNBC, significant knowledge gaps exist. Robust evidence shows that young age at breast cancer diagnosis indicates a distinct entity; however, the prevalence, oncologic outcomes, and treatment modalities of young age breast cancer vary and remain controversial.

We conducted this population-based study of eTNBC with BRCA genetic testing results in an attempt to better define the therapeutic schedule of BRCA-associated eTNBC and the effect of germline mutations on the clinicopathologic features and outcomes of these tumors.

## Materials and Methods

### Study Population and Ethical Statement

A retrospective review was conducted to identify patients with unilateral invasive eTNBC (age at diagnosis ≤40 years) who underwent surgery at Fudan University Shanghai Cancer Center between 2012 and 2018. The following variables were collected: genetic data (BRCA genetic test results), clinicopathologic data (age at diagnosis, family history of breast cancer or ovarian cancer (FH of BC or OC) in first- or second-degree relatives, parity, body mass index (BMI), histopathology, nuclear grade, tumor size, lymph node involvement and proliferative index), and treatment data (surgical type and adjuvant systemic therapy according to local protocols). Patients with a previous invasive breast cancer or ductal carcinoma *in situ* or bilateral breast cancer were excluded. This study was approved by the Ethical Committee of the Shanghai Cancer Center of Fudan University.

### BRCA Mutation Analysis

Briefly, genomic DNA extracted from peripheral blood was subjected to next-generation sequencing (NGS) according to the manufacturer’s instruction. All mutations considered disease-associated were confirmed through Sanger sequencing. The details of procedures of NGS and interpretation of the mutations were described in our previous study (20), and the genetic testing results were available before decision-making.

### Outcome Measures and Statistical Analysis

Overall survival (OS) was defined as the time from surgery to death from any cause. Disease-free survival (DFS) was defined as the interval from definitive surgery to any recurrence, contralateral breast cancer, distant metastasis, or death irrespective of cause. Breast cancer-specific survival (BCSS) was defined as the interval of survival time from surgery to death caused by breast cancer. Survivals were estimated using the Kaplan–Meier method. The log-rank test was adopted to compare survival outcomes between different conditions of patients. Categorical variables were compared using Pearson’s chi-squared test or Fisher’s exact test, and continuous variables were compared using independent t-test, as appropriate. Hazard ratios (HRs) and 95% confidential intervals (CIs) for univariate and multivariate analyses were calculated using Cox proportional hazards models. All tests were two-sided, and P <0.05 was considered statistically significant. Statistical analysis was performed using SPSS for Windows (version 23.0, SPSS Inc., Chicago, IL, USA).

## Results

### Patient Demographics and Clinicopathologic Characteristics

A total of 355 eTNBC patients were eligible and subjected to this analysis, of whom 67 (18.87%) patients were BRCA mutated and 288 (81.13%) patients were BRCA wild. Of the 67 patients with BRCA mutations, 58 (86.57%) had BRCA1 mutations and nine (13.43%) BRCA2 (data not shown). The prevalence of BRCA mutations with respect to patient demographics and clinicopathologic characteristics is presented in **Table 1**. Median age at diagnosis was 34 years (range, 24–40 years) and 35 years (range, 21–40 years) for BRCA mutated and BRCA wild eTNBC patients, respectively. No statistical significance in the proportion of FH of BC or OC in first- or second-degree relatives was exhibited (17.91 and 14.58% of BRCA mutated and BRCA wild subgroups, respectively). BMI, full-term pregnancy, lymph node involvement, and proliferative index were not predictive of BRCA mutation status.

**Table 1:** Baseline and treatment features of eTNBC patients grouped by germline BRCA status.

Characteristic	BRCA^mut^ (n = 67)		BRCA^wt^ (n = 288)		*P*-value
	No.	%	No.	%	
Age at diagnosis (years)					
Median	34		35		
Range	24–40		21–40		
BMI (kg/m^2^)					0.95
<18.5	3	4.48	20	6.94	
18.5–24.9	50	74.63	207	71.88	
≥25	11	16.42	48	16.67	
Unknown	3	4.48	13	4.51	
FH of BC or OC					0.62
Yes	12	17.91	42	14.58	
No	55	82.09	246	85.42	
Parity					0.62
Yes	57	85.07	235	81.60	
No	10	14.93	53	18.40	
Histology					0.90
IDC	61	91.04	257	89.24	
Mixed	5	7.46	25	8.68	
Other/Unknown	1	1.49	6	2.08	
Size (mm)					0.07
pT1	32	47.76	89	30.90	
pT2	25	37.31	140	48.61	
pT3	1	1.49	12	4.17	
Unknown	9	13.43	47	16.32	
Nodes					0.41
0	43	64.18	154	53.47	
1–3	19	28.36	86	29.86	
4–9	4	5.97	28	9.72	
≥10	1	1.49	16	5.56	
Unknown	0	0.00	4	1.39	
Stage					**0.01**
I	23	34.33	50	17.36	
II	30	44.78	146	50.69	
III	5	7.46	44	15.28	
Unknown	9	13.43	48	16.67	
Histological grade					0.08
I and II	4	5.97	39	13.54	
III	55	82.09	200	69.44	
Unknown	8	11.94	49	17.01	
Ki-67 (%)					0.11
<20	1	1.49	9	3.13	
≥20	62	92.54	237	82.29	
Unknown	4	5.97	42	14.58	
Surgery					0.90
BCS	27	40.30	123	42.71	
Mastectomy	40	59.70	165	57.29	
Reconstruction					0.47
Yes	7	10.45	20	6.94	
No	60	89.55	268	93.06	
Radiation therapy					0.30
Yes	39	58.21	187	64.93	
No	25	37.31	81	28.13	
Unknown	3	4.48	20	6.94	
Adjuvant chemotherapy					**0.04**
Anthracyclines/Taxanes	54	80.60	180	62.50	
Anthracyclines	4	5.97	39	13.54	
Taxanes	4	5.97	26	9.03	
Others	5	7.46	43	14.93	

BMI, body mass index; FH of BC or OC, family history of breast cancer or ovarian cancer. pT1, pathological tumor size ≤2 cm; pT2, 2 cm <pathological tumor size ≤5 cm; pT3, pathological tumor size >5 cm; IDC, infiltrating ductal carcinoma; Nuclear grade I, well differentiated; Nuclear grade II, moderate differentiation; Nuclear grade III, poor differentiation; Ki-67, cell proliferation index; BCS, breast conserving surgery.

The bold values suggested the differences are of statistical significance between the indicated groups.

Predictably, the vast majority of participants had infiltrating ductal carcinoma (IDC), followed by mixed pathological pattern, including IDC with invasive lobular carcinoma, IDC with medullary carcinoma, and IDC with ductal carcinoma *in situ*; all of those were similar between the two subgroups. Although there was a trend for BRCA mutated tumors to have higher histological grade than BRCA wild tumors, this did not reach statistical significance (*P* = 0.08). BRCA mutated tumors were more frequently treated with anthracyclines and taxane-based combination chemotherapy (*P* = 0.04). However, similar likelihoods of receiving radiation therapy and breast reconstruction were manifested in both subgroups.

### Survival Estimates

**Figure 1:**
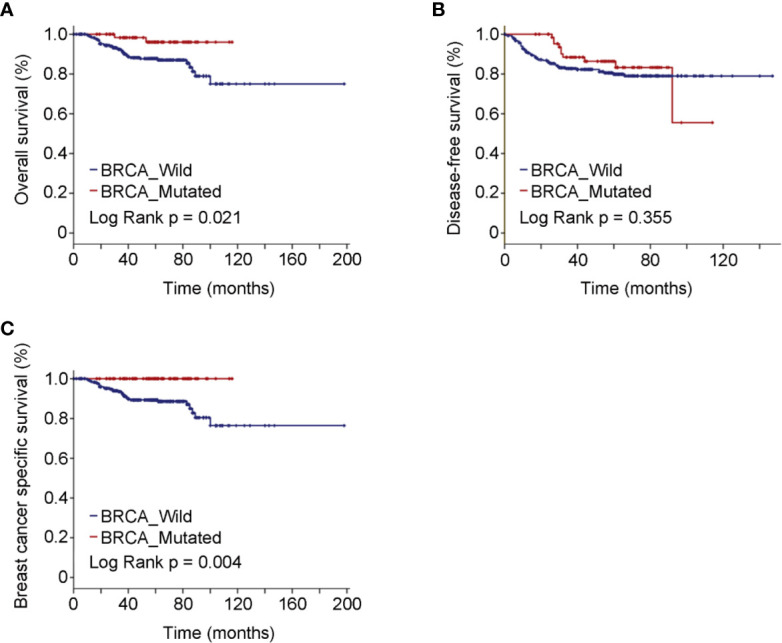
Oncologic outcomes for all patients enrolled in the study cohort by BRCA mutation status. **(A)** Kaplan–Meier estimates of OS. **(B)** Kaplan–Meier estimates of DFS. **(C)** Kaplan–Meier estimates of BCSS.

Median follow-up of the study cohort was 56.5 months. A total of 38 deaths, 32 breast cancer-specific events, and 68 deaths or recurrences were observed. The estimated OS was significantly better in BRCA mutated eTNBC patients than BRCA wild ones (*P* = 0.021). Although limitations of the retrospective study and some missing values of variables existed, the Cox regression analyses indicated that only BRCA status [HR = 0.22, 95%CI(0.05–0.91)] was the independent factor contributing the difference of OS outcome (data not shown). The same tendency was demonstrated with regard to BCSS (*P* = 0.004). As to DFS, there was no significant difference between BRCA mutated patients and BRCA wild ones (*P* = 0.355). The Kaplan–Meier plots for OS, DFS, and BCSS by mutational status are shown in **Figure 1A–C**. Though no statistical significance was obtained in landmark analysis at the 5-year time point, the trend was absolutely reversed.

### Treatment Interventions

**Figure 2:**
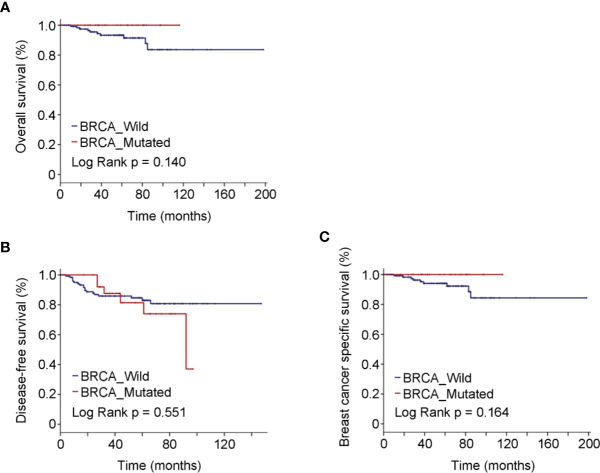
Oncologic outcomes for patients who underwent BCS by BRCA mutation status. **(A)** Kaplan–Meier estimates of OS. **(B)** Kaplan–Meier estimates of DFS. **(C)** Kaplan–Meier estimates of BCSS.

The most frequently used chemotherapy regimen was anthracyclines with or without taxanes, but quite remarkably, no participant was receiving platinum-containing regimen (**Table 1**). Breast-conserving surgery (BCS) was performed in 40.30% BRCA mutated eTNBC patients and 42.71% BRCA wild patients. The clinicopathologic features of eTNBC patients who underwent BCS were parallel between subgroups divided by BRCA mutation status (**Table 2**). Clinical outcomes, whether OS, DFS, or BCSS, did not significantly differ between the two subgroups (**Figure 2**).

**Table 2:** Clinicopathologic features of eTNBC patients underwent BCS grouped by germline BRCA1 status.

Characteristic	BRCA^mut^ (n = 27)		BRCA^wt^ (n = 123)		*P*-value
	No.	%	No.	%	
Age at diagnosis (years)					
Median	32		34		
Range	24–40		21–40		
BMI (kg/m^2^)					0.66
<18.5	1	3.70	5	4.07	
18.5–24.9	18	66.67	92	74.80	
≥25	6	22.22	21	17.07	
Unknown	2	7.41	5	4.07	
FH of BC or OC					
Yes	3	11.11	21	17.07	0.57
No	24	88.89	102	82.93	
Parity					0.41
Yes	23	85.19	93	75.61	
No	4	14.81	30	24.39	
Histology					0.87
IDC	25	92.59	108	87.80	
Mixed	2	7.41	10	8.13	
Other/Unknown	0	0.00	5	4.07	
Size (mm)					0.21
pT1	13	48.15	37	30.08	
pT2	9	33.33	59	47.97	
pT3	0	0.00	0	0.00	
Unknown	5	18.52	27	21.95	
Nodes					0.68
0	19	70.37	75	60.98	
1–3	8	29.63	34	27.64	
4–9	0	0.00	9	7.32	
≥10	0	0.00	2	1.63	
Unknown	0	0.00	3	2.44	
Stage					0.27
I	9	33.33	24	19.51	
II	13	48.15	61	49.59	
III	0	0.00	10	8.13	
Unknown	5	18.52	28	22.76	
Histological grade					0.94
I and II	3	11.11	13	10.57	
III	20	74.07	94	76.42	
Unknown	4	14.81	16	13.01	
Ki-67 (%)		0.00			0.90
<20	0		4	3.25	
≥20	24	88.89	101	82.11	
Unknown	3	11.11	18	14.63	
Reconstruction					1.00
Yes	1	3.70	4	3.25	
No	26	96.30	119	96.75	
Radiation therapy					0.99
Yes	22	81.48	101	82.11	
No	3	11.11	14	11.38	
Unknown	2	7.41	8	6.50	
Adjuvant chemotherapy					0.57
Anthracyclines/Taxanes	21	77.78	82	66.67	
Anthracyclines	1	3.70	12	9.76	
Taxanes	1	3.70	13	10.57	
Others	4	14.81	16	13.01	

BMI, body mass index; FH of BC or OC, family history of breast cancer or ovarian cancer; pT1, pathological tumor size ≤2 cm; pT2, 2 cm <pathological tumor size ≤5 cm; pT3, pathological tumor size >5cm; IDC, infiltrating ductal carcinoma; Nuclear grade I, well differentiated; Nuclear grade II, moderate differentiation; Nuclear grade III, poor differentiation; Ki-67, cell proliferation index. BCS, breast conserving surgery.

**Figure 3:**
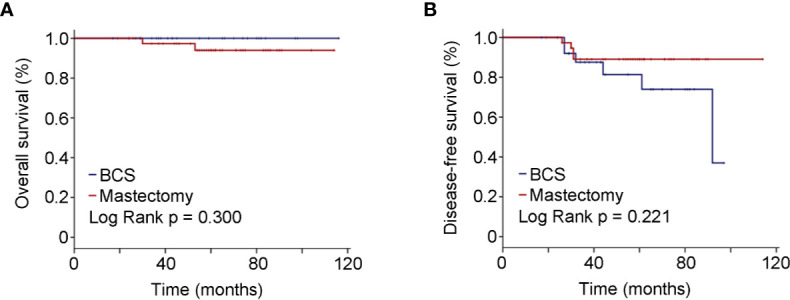
Oncologic outcomes for patients with BRCA positivity by surgical schedules. **(A)** Kaplan–Meier estimates of OS. **(B)** Kaplan–Meier estimates of DFS.

As to the 67 BRCA mutated eTNBC patients grouped by surgical treatment, 27 underwent BCS, and 40 chose mastectomy. Unsurprisingly, radiation therapy was significantly more frequent in those who underwent BCS. Beyond that, basic characteristics were comparable between the two subgroups, as shown in **Table 3**. Likewise, OS and DFS were not significantly different between the two subgroups (**Figure 3**). BCSS was not analyzed because of few events and unrepresented statistical power.

**Table 3:** Clinicopathologic features of BRCA^mut^ eTNBC patients grouped by surgical treatment.

Characteristic	BCS(n=27)		Mastectomy (n=40)		*P*-value
	No.	%	No.	%	
Age at diagnosis (years)					
Median	32		35		
Range	24–40		29–40		
BMI (kg/m^2^)					0.55
<18.5	1	3.70	2	5.00	
18.5–24.9	18	66.67	32	80.00	
≥25	6	22.22	5	12.50	
Unknown	2	7.41	1	2.50	
FH of BC or OC					0.33
Yes	3	11.11	9	22.50	
No	24	88.89	31	77.50	
Parity					1.00
Yes	23	85.19	34	85.00	
No	4	14.81	6	15.00	
Histology					0.99
IDC	25	92.59	36	90.00	
Mixed	2	7.41	3	7.50	
Other/Unknown	0	0.00	1	2.50	
Size (mm)					0.73
pT1	13	48.15	19	47.50	
pT2	9	33.33	16	40.00	
pT3	0	0.00	1	2.50	
Unknown	5	18.52	4	10.00	
Nodes					0.37
0	19	70.37	24	60.00	
1–3	8	29.63	11	27.50	
4–9	0	0.00	4	10.00	
≥10	0	0.00	1	2.50	
Stage					0.24
I	9	33.33	14	35.00	
II	13	48.15	17	42.50	
III	0	0.00	5	12.50	
Unknown	5	18.52	4	10.00	
Histological grade					0.30
I and II	3	11.11	1	2.50	
III	20	74.07	35	87.50	
Unknown	4	14.81	4	10.00	
Ki-67 (%)					0.29
<20	0	0.00	1	2.50	
≥20	24	88.89	38	95.00	
Unknown	3	11.11	1	2.50	
Reconstruction					0.23
Yes	1	3.70	6	15.00	
No	26	96.30	34	85.00	
Radiation therapy					**<0.01**
Yes	22	81.48	17	42.50	
No	3	11.11	22	55.00	
Unknown	2	7.41	1	2.50	
Adjuvant chemotherapy					0.27
Anthracyclines/Taxanes	21	77.78	33	82.50	
Anthracyclines	1	3.70	3	7.50	
Taxanes	1	3.70	3	7.50	
Others	4	14.81	1	2.50	

BMI, body mass index; FH of BC or OC, family history of breast cancer or ovarian cancer; pT1, pathological tumor size ≤2 cm; pT2, 2 cm <pathological tumor size ≤5 cm; pT3, pathological tumor size >5 cm; IDC, infiltrating ductal carcinoma; Nuclear grade I, well differentiated; Nuclear grade II, moderate differentiation; Nuclear grade III, poor differentiation; Ki-67, cell proliferation index; BCS, breast conserving surgery.

## Discussion

In this study, we investigated the clinical and pathological characteristics as well as survival outcomes in a cohort of unselected women with eTNBC patients and to what extent these phenotypes could be contributed to BRCA mutation. The prevalence of BRCA mutation in this study was 18.87%, consistent with previous reports mainly based on institutional- or hospital-based samples (21–26). Published reports have indicated that a high BMI was protective against breast cancer risk in premenopausal women (27), and no significant differences in BMI, warning that a higher BMI may be favorable in BRCA mutated eTNBC patients. It was worth noting that FH of BC or OC seemed lower in the current study as opposed to previous reports (22, 25), which suggested 19% or even 90% in young age breast cancer. Generally, BRCA mutated patients were more likely to have a FH of BC or OC than BRCA wild patients (28–30). The fact that patients self-reported family history might not be accurate, resulting in underrepresentation. Additionally, ethnicity and geographical distribution should be taken into consideration.

As to disease characteristics, of note, in agreement with most previous studies (21–23, 25), the tumor burden in BRCA mutated eTNBC patients was comparable with that in BRCA wild ones. Recently, a prospective large cohort study showed no significant difference in tumor size between BRCA mutated and BRCA wild young-onset breast cancer (26). More recently, a retrospective study conducted in Chinese early-onset breast cancer with more than 80% TNBC also failed to demonstrate a significant difference of tumor size (31). Herein, the other clinicopathologic features were in broad agreement with previous reports (23, 32–34), indicating that eTNBC tends to be aggressive irrespective of BRCA mutation.

Although the prognosis of eTNBC patients with BRCA mutation was inclusive, numerous studies failed to document an inferior survival outcome of eTNBC patients carrying BRCA mutation compared with their counterparts (35, 36). Confirming the previous studies (26, 37, 38), OS and BCSS of eTNBC patients with BRCA mutation were superior to those of their BRCA wild counterparts; however, DFS was not significantly different between the two subgroups. It should be noted that greater sensitivity to adjuvant systemic therapy was probable the key factor resulting in better survival outcomes, under the circumstance of equality of disease characteristics and stage at diagnosis in the current study. Up to now, the optimal treatment for eTNBC patients with BRCA mutation was largely unknown and remained a matter of debate. Interestingly, in view of patients underwent BCS, the survival outcomes were similar between the two subgroups with similar baseline traits. Simultaneously, the same tendency was revealed in patients with BRCA mutated eTNBC, no matter what surgical procedures were performed. In recent decades, although the risk for recurrent breast cancer or contralateral breast cancer was higher in BRCA mutated TNBC, advances in biology and systemic therapy have decreased the risk to an acceptable level (39–42). Thus, it was rational to propose that BCS was a safe and feasible option for patients with BRCA-associated eTNBC if systemic therapy was available and tolerable.

The current study has some strengths. BRCA genetic testing was performed in all participants to avoid referral bias, and the testing results were available to physicians in decision-making treatment. Furthermore, the comprehensive details of demographic and clinicopathologic characteristics were derived from medical records to reduce the potential bias induced by using questionnaires. One limitation of this study was that the FH was obtained from individual report rather than medical records. Another limitation of this study was that the total number of the study cohort was small, especially of the BRCA2 positive subgroup, meaning we were unable to distinguish the effect of BRCA1 on prognosis from that of BRCA2. Besides, the contribution of other factors, such as neoadjuvant therapies, prophylactic surgeries, on the outcomes were unavailable. Future studies that recruit larger sample sizes with precise FH are needed to offer an extrapolative conclusion.

In conclusion, our results suggested that eTNBC patients with BRCA mutations tend to have better OS and BCSS, which might be attributed to more benefit from systemic therapy. The BCS procedure would be a safe alternative surgical option to early-stage BRCA mutation eTNBC patients on condition that systemic therapy was available and tolerable. Future studies with large size and comprehensive clinicopathologic details are urged to validate whether BRCA mutation patients are more sensitive to chemotherapy.

## Data Availability Statement

The raw data supporting the conclusions of this article will be made available by the authors, without undue reservation.

## Ethics Statement

The studies involving human participants were reviewed and approved by the Ethical Committee of the Shanghai Cancer Center of Fudan University. The patients/participants provided their written informed consent to participate in this study.

## Author Contributions

AC conceptualized and designed the study. FY and AC provided the study patients and methods. FY, MH, LH, GL, XH, ZS, GD and AC collected the data. FY and AC analyzed and interpreted the data. FY and AC wrote the manuscript. FY, MH, LH, GL, XH, ZS, GD and AC gave the final approval of the manuscript. All authors contributed to the article and approved the submitted version.

## Funding

The authors are grateful for the financial support of the Shanghai Science and Technology Innovation Action Plan (20ZR1412000).

## Conflict of Interest

The authors declare that the research was conducted in the absence of any commercial or financial relationships that could be construed as a potential conflict of interest.
